# Development and validation of a nomogram for predicting the survival of patients with non-metastatic nasopharyngeal carcinoma after curative treatment

**DOI:** 10.1186/s40880-016-0160-9

**Published:** 2016-11-25

**Authors:** Wenhua Liang, Guanzhu Shen, Yaxiong Zhang, Gang Chen, Xuan Wu, Yang Li, Anchuan Li, Shiyang Kang, Xi Yuan, Xue Hou, Peiyu Huang, Yan Huang, Hongyun Zhao, Ying Tian, Chong Zhao, Li Zhang

**Affiliations:** 1 State Key Laboratory of Oncology in South China, Collaborative Innovation Center for Cancer Medicine, Sun Yat-sen University Cancer Center, Guangzhou, Guangdong 510060 P. R. China; 2 Department of Medical Oncology, Sun Yat-sen University Cancer Center, 651 Dongfeng Road East, Guangzhou, Guangdong 510060 P. R. China; 3 Department of Radiation Oncology, Sun Yat-sen University Cancer Center, Guangzhou, Guangdong 510060 P. R. China; 4 Department of Radiotherapy, Cancer Hospital of Guangzhou Medical University, Guangzhou, Guangdong 510095 P. R. China; 5 Department of Thoracic Surgery/Oncology, The First Affiliated Hospital of Guangzhou Medical University, Guangzhou, Guangdong 510120 P. R. China; 6 Department of Radiotherapy, Sun Yat-sen University Cancer Center, 651 Dongfeng Road East, Guangzhou, Guangdong 510060 P. R. China

**Keywords:** Nasopharyngeal carcinoma, Nomogram, Prognosis

## Abstract

**Background:**

The TNM staging system is far from perfect in predicting the survival of individual cancer patients because only the gross anatomy is considered. The survival rates of the patients who have the same TNM stage disease vary across a wide spectrum. This study aimed to develop a nomogram that incorporates other clinicopathologic factors for predicting the overall survival (OS) of non-metastatic nasopharyngeal carcinoma (NPC) patients after curative treatments.

**Methods:**

We retrospectively collected the clinical data of 1520 NPC patients who were diagnosed histologically between November 2000 and September 2003. The clinical data of a separate cohort of 464 patients who received intensity-modulated radiation therapy (IMRT) between 2001 and 2010 were also retrieved to examine the extensibility of the model. Cox regression analysis was used to identify the prognostic factors for building the nomogram. The predictive accuracy and discriminative ability were measured using the concordance index (c-index).

**Results:**

We identified and incorporated 12 independent clinical factors into the nomogram. The calibration curves showed that the prediction of OS was in good agreement with the actual observation in the internal validation set and IMRT cohort. The c-index of the nomogram was statistically higher than that of the 7th edition TNM staging system for predicting the survival in both the primary cohort (0.69 vs. 0.62) and the IMRT cohort (0.67 vs. 0.63).

**Conclusion:**

We developed and validated a novel nomogram that outperformed the TNM staging system in predicting the OS of non-metastatic NPC patients who underwent curative therapy.

## Background

Nasopharyngeal carcinoma (NPC) is relatively common among patients living in the Far East and their descendants who have migrated abroad [1, 2]. NPC in these populations is associated with Epstein–Barr virus (EBV) infection, which is rare in NPC patients in other parts of the world [3]. EBV-associated NPC has an increased tendency to metastasize to regional lymph nodes and distant sites. Radiotherapy remains the backbone of care in non-metastatic NPC patients, along with various combinations with chemotherapy, including induction, concomitant, and adjuvant chemotherapy [4].

To date, the American Joint Committee on Cancer (AJCC) TNM classification has been the most widely used staging system to estimate the prognosis and guide treatment options [5]. However, the TNM staging system is far from perfect because it only considers the tumor size and extension (T stage) and node involvement (N stage) without considering other factors with prognostic values, such as clinicopathologic factors, treatment-related factors, and tumor markers. In addition, the patient survival is significantly altered after curative therapy, such as surgery and radiotherapy. Therefore, a more accurate prediction of the survival is required in clinical practice.

In recent years, a novel prognostic model, called a nomogram, has proved a reliable model for cancer prognosis prediction [6–8]. Nomograms incorporate assessable variables through weighing their respective significance to the survival and function as a simple tool for individual survival prediction [9]. However, no nomograms have been developed for NPC. Therefore, based on a large cohort in our center, we aimed to establish a nomogram for individual survival prediction of NPC patients without distant metastasis who had undergone curative therapy. In addition, a cohort of patients who received intensity-modulated radiation therapy (IMRT) was also included for validation to test whether this nomogram could be applied to predict survival of these patients.

## Patients and methods

### Patient selection and data processing

Patients with histologically proven NPC treated between November 2000 and September 2003 at Sun Yat-sen University Cancer Center were examined. The patients with AJCC stage I–IVb NPC who had undergone curative treatments (including radiotherapy alone and radiotherapy in combination with either adjuvant chemotherapy or neoadjuvant chemotherapy or both) were included. An independent cohort of patients who underwent IMRT at the same institution between February 2001 and August 2010 was also included. Patients who had distant metastasis or missing data on important variables were excluded. Ethical approval was obtained from our center through the Institutional Review Board.

Clinical data were retrieved, including the age; sex; history of smoking and alcohol consumption; pathologic data [histological type and pathologic tumor (T), node (N), and metastasis (M) statuses]; treatment-related factors (radiation dose and access to neoadjuvant, concomitant, or adjuvant chemotherapy); and serological factors [hemoglobin count, platelet count, neutrophil count, lymphocyte count, neutrophil to lymphocyte ratio (NLR), titers of immunoglobulin A against Epstein–Barr virus viral capsid antigen (VCA-IgA), immunoglobulin A against Epstein–Barr virus viral early antigen (EA-IgA), and anti-DNase, and serum levels of lactate dehydrogenase (LDH) and alkaline phosphatase (ALP)].

Follow-up data for all patients were obtained from their most recent medical review, which consisted of scheduled clinical examination and assessment of chest X-ray photograms, chest or abdominal computed tomography scans, and head and neck magnetic resonance imaging every 3 months during the first 2 years after primary treatment and every 6 months thereafter, as well as the survival status every 3 months, which was evaluated by the follow-up team. The last follow-up was carried out in October 2014. Clinical staging was performed according to the Union for International Cancer Control (UICC)/American Joint Committee on Cancer (AJCC) TNM staging system (2009 version).

Continuous variables were transformed into categorical variables, and the cutoffs of all variables were recognized with the largest χ^2^ value in the log-rank test using a Cut-Point Optimization Tool (X-Tile, Yale University, New Haven, CT, USA) [10]. The normal range of body mass index (BMI) was set at 20–26 kg/m^2^. The cutoff values for other variables were derived using X-Tile, and they were discussed and confirmed by clinical expertise as follows: age (50 years), hemoglobin (150 g/L), NLR (3.5), platelet count (300 × 10^9^/L), ALP level (90 U/L), VCA-IgA titer (1:1280), EA-IgA titer [0, (negative vs. positive)], and anti-DNase titer (50%).

### Construction and validation of the nomogram

Statistical analyses to identify independent prognostic factors were conducted with SPSS 17.0 for Windows (SPSS, Chicago, IL, USA). Considering the large sample size of this cohort and the importance of independent validation, we adopted a data-splitting method using Rv.Uniform function in SPSS to randomly assign 80% of the patients to the training set for nomogram construction and 20% to the internal validation set. Overall survival (OS) was calculated from the date of diagnosis. The OS curves were generated using the Kaplan–Meier method and were compared using the log-rank test. Covariates achieving significance at a level of *P* < 0.05 were entered into the Cox regression model for multivariate analyses. Based on the results from multivariate analysis, a nomogram was formulated using R2.14.1 (http://www.r-project.org/) with the survival and rms package, which was based on the theory by Harrel et al. [11]. A final model was selected using a backward stepdown process, which incorporated Akaike’s information criterion as the stopping rule [12].

The derived scores were divided into the 25th, 50th, 75th, and 100th percentile to subgroup the patients. Calibration of the nomogram for the 1-, 3-, and 5-year OS was performed by comparing the median predicted OS with the actual OS from observed Kaplan–Meier estimates. The model performance for predicting the outcome was evaluated by calculating the concordance index (c-index) [13]. The value of the c-index ranged from 0.5 to 1.0, which indicates random chance to a perfect ability to correctly discriminate between the outcome and model.

### Performance of the nomogram beyond the TNM staging system

We sought to evaluate the independent discrimination ability of the nomogram beyond the standard TNM staging. Kaplan–Meier OS curves of patient subgroups were delineated.

## Results

### Clinicopathologic characteristics of patients in the primary cohort

A total of 1520 patients with stage I–IVb NPC who had undergone at least radiotherapy in the primary cohort were eligible for final analysis, with 1036 deaths in a median follow-up of 86.6 months (range 1.4–115.0 months). The predominant histological type was World Health Organization (WHO) type III. Most of the included patients received conventional radiotherapy. Of the 1520 patients, 1216 (80%) were assigned to the training set, and 304 (20%) to the internal validation set. An independent cohort of 464 patients who underwent IMRT was selected, with 376 events in a median follow-up of 86.1 months (range 29.2–133.0 months). The clinicopathologic features of patients in the training set, internal validation set, and IMRT cohort are summarized in Table 1.

**Table 1:** Demographic and clinicopathologic characteristics of non-metastatic nasopharyngeal carcinoma patients in the training set, internal validation set, and IMRT cohort

Variable	Primary cohort [cases (%)]			IMRT cohort [cases (%)]
	Total	Training set	Validation set	
Total	1520	1216	304	464
Sex				
Male	1161 (76.4)	922 (75.8)	239 (78.6)	361 (77.8)
Female	359 (23.6)	294 (24.2)	65 (21.4)	103 (22.2)
Age (years)				
<50	977 (64.3)	769 (63.2)	208 (68.4)	345 (74.4)
≥50	543 (35.7)	447 (36.8)	96 (31.6)	119 (25.6)
Smoking history				
No	789 (51.9)	630 (51.8)	159 (52.3)	271 (58.4)
Yes	731 (48.1)	586 (48.2)	145 (47.7)	193 (41.6)
Alcohol consumption				
No	1282 (84.3)	1030 (84.7)	252 (82.9)	387 (83.4)
Yes	238 (15.7)	186 (15.3)	52 (17.1)	77 (16.6)
BMI (kg/m^2^)				
>26	210 (13.8)	174 (14.3)	36 (11.8)	150 (32.3)
20–26	949 (62.4)	755 (62.1)	194 (63.8)	312 (67.2)
<20	361 (23.8)	287 (23.6)	74 (24.3)	2 (0.4)
Hemoglobin level (g/L)				
>150	498 (32.8)	399 (32.8)	99 (32.6)	186 (40.1)
≤150	1022 (67.2)	817 (67.2)	205 (67.4)	278 (59.9)
NLR				
<3.5	1129 (74.3)	906 (74.5)	223 (73.4)	384 (82.8)
≥3.5	391 (25.7)	310 (25.5)	81 (26.6)	80 (17.2)
Platelet count (×10^9^/L)				
<300	1251 (82.3)	1000 (82.2)	251 (82.6)	408 (87.9)
≥300	269 (17.7)	216 (17.8)	53 (17.4)	56 (12.1)
LDH level (U/L)				
<190	897 (59.0)	717 (59.0)	180 (59.2)	352 (75.9)
190–240	399 (26.3)	315 (25.9)	84 (27.6)	80 (17.2)
>240	224 (14.7)	184 (15.1)	40 (13.2)	32 (6.9)
ALP level (U/L)				
<90	1227 (80.7)	978 (80.4)	249 (81.9)	401 (86.4)
≥90	293 (19.3)	238 (19.6)	55 (18.1)	63 (13.6)
Histological type				
WHO I	6 (0.4)	5 (0.4)	1 (0.3)	1 (0.2)
WHO II	102 (6.7)	78 (6.4)	24 (7.9)	41 (8.8)
WHO III	1412 (92.9)	1133 (93.2)	279 (91.8)	422 (90.9)
T stage				
T1	262 (17.2)	198 (16.3)	64 (21.1)	86 (18.5)
T2	746 (49.1)	603 (49.6)	143 (47.0)	119 (25.6)
T3	267 (17.6)	213 (17.5)	54 (17.8)	191 (41.2)
T4	245 (16.1)	202 (16.6)	43 (14.1)	68 (14.7)
N stage				
N0	419 (27.6)	337 (27.7)	82 (27.0)	116 (25.0)
N1	642 (42.2)	510 (41.9)	132 (43.4)	193 (41.6)
N2	408 (26.8)	326 (26.8)	82 (27.0)	144 (31.0)
N3	51 (3.4)	43 (3.5)	8 (2.6)	11 (2.4)
TNM stage				
I	79 (5.2)	60 (4.9)	19 (6.3)	43 (9.3)
II	638 (42.0)	511 (42.0)	127 (41.8)	113 (24.4)
III	515 (33.9)	408 (33.6)	107 (35.2)	231 (49.8)
IV	288 (18.9)	237 (19.5)	51 (16.8)	77 (16.6)
VCA-IgA titer				
<1:1280	1275 (83.9)	1013 (83.3)	262 (86.2)	420 (90.5)
≥1:1280	245 (16.1)	203 (16.7)	42 (13.8)	44 (9.5)
EA-IgA				
Negative	334 (22.0)	272 (22.4)	62 (20.4)	127 (27.4)
>0	1186 (78.0)	944 (77.6)	242 (79.6)	337 (72.6)
Anti-DNase (%)				
≤50	516 (33.9)	407 (33.5)	109 (35.9)	221 (47.6)
>50	1004 (66.1)	809 (66.5)	195 (64.1)	205 (44.2)^a^
Neoadjuvant chemotherapy				
Yes	997 (65.6)	800 (65.8)	197 (64.8)	304 (65.5)
No	523 (34.4)	416 (34.2)	107 (35.2)	160 (34.5)
Concomitant chemotherapy				
Yes	1171 (77.0)	933 (76.7)	238 (78.3)	145 (31.3)
No	349 (23.0)	283 (23.3)	66 (21.7)	319 (68.7)
Adjuvant chemotherapy				
Yes	1513 (99.5)	1210 (99.5)	303 (99.7)	445 (95.9)
No	7 (0.5)	6 (0.5)	1 (0.3)	19 (4.1)
Radiation dose (Gy)				
≤75	1329 (87.4)	1059 (87.1)	270 (88.8)	240 (51.7)
>75	191 (12.6)	157 (12.9)	34 (11.2)	224 (48.3)

*IMRT* intensity-modulated radiation therapy; *BMI* body mass index; *NLR* neutrophil–lymphocyte ratio; *LDH* lactate dehydrogenase; *ALP* alkaline phosphatase; *WHO* World Health Organization; *VCA-IgA* immunoglobulin A against Epstein–Barr virus viral capsid antigen; *EA-IgA* immunoglobulin A against Epstein–Barr virus viral early antigen

^a^The data of 38 patients were missing

### Independent prognostic factors in the training set

The data from the training set were used to identify prognostic factors and build the model. The results of the univariate analysis are listed in Table 2. Male gender, over 50 years old, smokers, drinkers, increased NLR (≥3.5), increased platelet count (≥300 × 10^9^/L), increased ALP level (≥90 U/L), positive EA-IgA, high anti-DNase titer (>50%), receiving no neoadjuvant, concomitant, or adjuvant chemotherapy, and high radiation dose (>75 Gy) were all associated with poor prognosis. Baseline anemia and a high titer of VCA-IgA were probably associated with poor prognosis, but the differences were not significant. Both the pathologic T and N stages had a significant impact on OS; there was an increased risk for deaths as the stage increased. The BMI and LDH were divided into three categories. A high BMI indicated a favorable survival outcome, whereas a reverse trend was revealed for the LDH level. WHO type III was the predominant histological type and associated with the best prognosis among all three types.

**Table 2:** Cox univariate and multivariate analyses of prognostic factors for the overall survival of non-metastatic nasopharyngeal carcinoma patients after treatment

Variable	Univariate analysis	Multivariate analysis		
	*P* value	HR	95% CI	*P* value
Sex	0.023	1.228	1.093–1.363	0.163
Age	<0.001	1.572	1.478–1.666	<0.001
Smoking history	0.025	0.957	0.845–1.070	0.833
Alcohol consumption	0.048	1.056	0.930–1.182	0.279
BMI	0.014	1.164	1.087–1.241	0.004
Hemoglobin	0.079	Not included in the multivariate analysis		
NLR	<0.001	1.324	1.225–1.423	0.001
Platelet count	0.015	1.165	1.048–1.282	0.366
LDH level	<0.001	1.332	1.273–1.391	<0.001
ALP level	0.001	1.235	1.126–1.344	0.185
Histological type	0.032	0.986	0.538–1.434	0.082
T stage	<0.001	1.264	1.212–1.316	<0.001
N stage	<0.001	1.457	1.399–1.515	<0.001
TNM stage	<0.001	Excluded due to collinearity		
VCA-IgA titer	0.099	Not included in the multivariate analysis		
EA-IgA	0.001	1.224	1.100–1.348	0.179
Anti-DNase titer	0.019	1.155	1.022–1.288	0.43
Neoadjuvant chemotherapy	<0.001	1.209	1.111–1.308	0.043
Concomitant chemotherapy	0.022	1.167	1.060–1.274	0.424
Adjuvant chemotherapy	0.035	1.747	1.238–2.256	0.37
Radiation dose	0.002	1.415	1.282–1.548	0.009

*HR* hazard ratio; *CI* confidence interval. Other abbreviations as in Table 1

Variables considered significant in the univariate analyses were entered in the Cox multivariate analysis. A total of 12 variables, including sex, age, T stage, N stage, BMI, NLR, radiation dose, assess to neoadjuvant or concomitant chemotherapy, EA-IgA titer, and serum LDH and ALP levels were proved independent in the multivariate Cox regression model and were incorporated in the nomogram according to the algorithm (Table 2).

### Prognostic nomogram for OS prediction

**Fig. 1:**
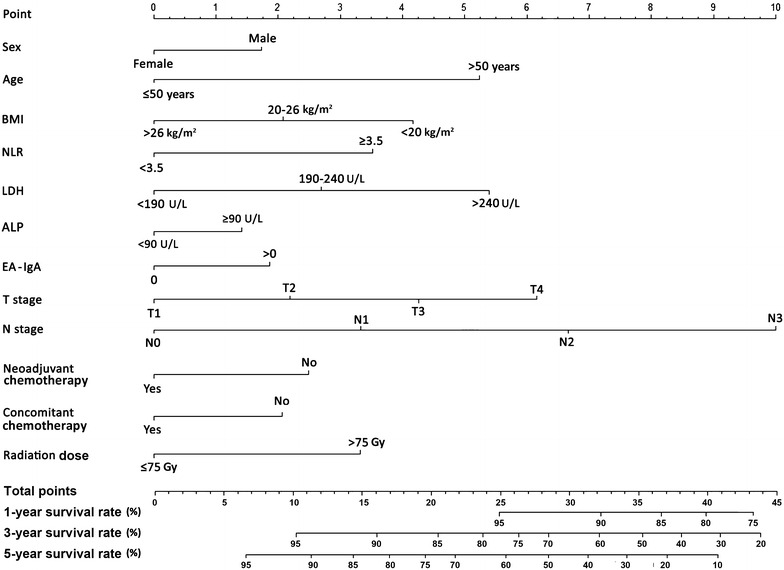
Prognostic nomogram for patients with non-metastatic nasopharyngeal carcinoma (NPC). *BMI* body mass index; *NLR* neutrophil–lymphocyte ratio; *LDH* lactate dehydrogenase; *ALP* alkaline phosphatase; *EA-IgA* immunoglobulin A against Epstein–Barr virus viral early antigen

Using the data of patients in the training set, a nomogram was constructed for OS prediction (Fig. 1). Longer lines indicate greater prognostic impact of specific variables, and larger points in the nomogram indicate shorter OS. The N stage had the greatest impact on OS, which was followed by the T stage, LDH level, and age. Subsequently, the BMI, NLR, and radiation dose were also found to be important. Furthermore, other independent prognostic factors provided additional prediction value.

Each subtype within the above variables was assigned a score on the point scale. By adding up the total score and locating it on the total point scale, we could easily draw a straight line down to determine the estimated probability of survival at each time point. The total scores ranged from 6 to 39. By dividing the range into five equal parts, we determined five risk subgroups of patients (scoring 0–12, 13–18, 19–24, 25–31, and ≥32).

### Validation of the nomogram

**Fig. 2:**
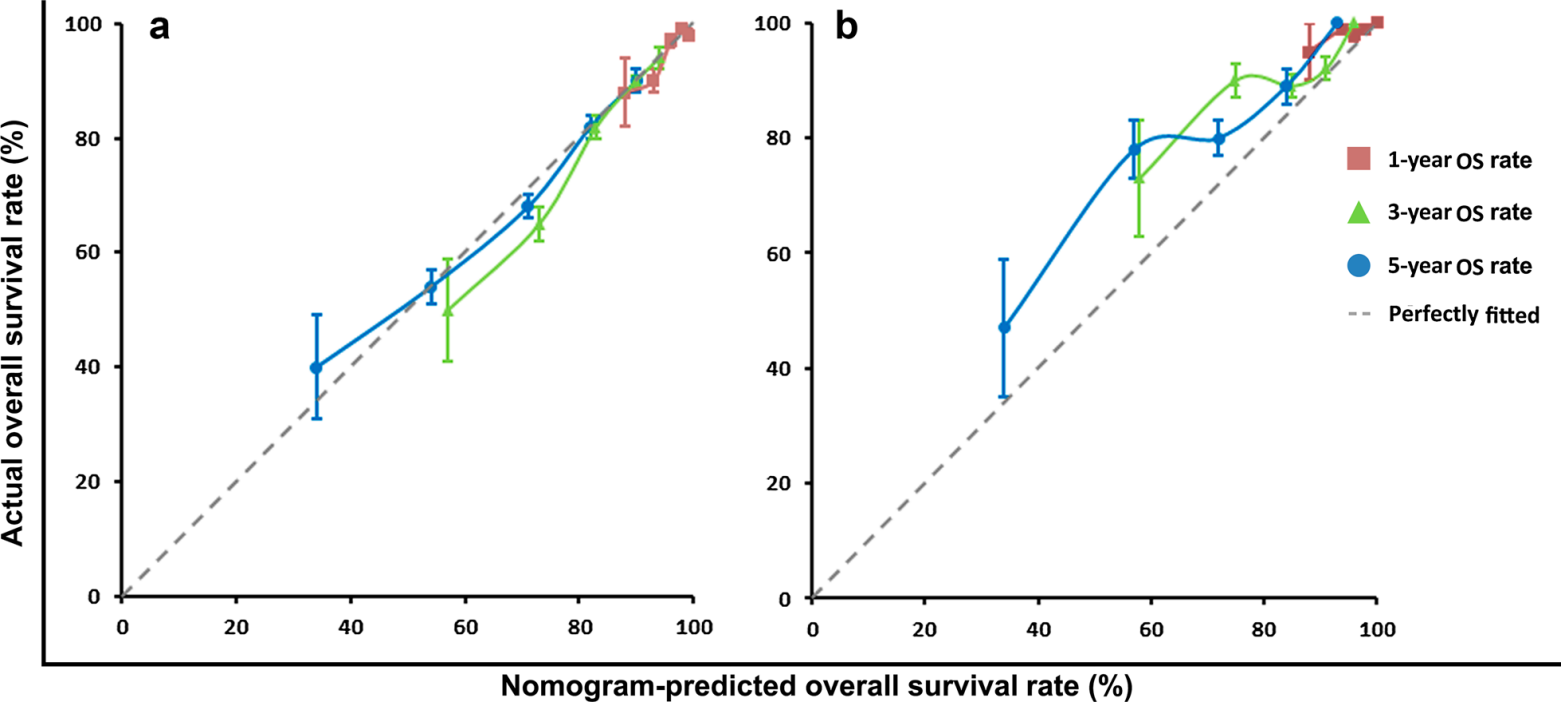
The calibration curve for predicting overall survival (OS) of patients with non-metastatic NPC in the internal validation set (**a**) and intensity-modulated radiation therapy (IMRT) cohort (**b**). The nomogram-predicted probability of OS is plotted on the x-axis; the actual OS is plotted on the y-axis

The data from the internal validation set were used to validate the model. The calibration plot based on the data from the internal validation set for the probability of OS at 1, 3, and 5 years demonstrated excellent agreement between the prediction according to the nomogram and actual observation (Fig. 2a). However, the predicted OS was slightly underestimated in the IMRT cohort (Fig. 2b). In addition, the Harrel’s c-index of the established nomogram to predict the OS of all patients in the primary cohort (combining the training set and the internal validation set) was significantly higher than that of the AJCC TNM staging system (0.69 [95% confidence interval {CI}, 0.67–0.71] vs. 0.62 [95% CI, 0.60–0.64], *P* = 0.003). With respect to OS prediction for the IMRT cohort, the c-index of the nomogram was also numerically higher than that of the TNM staging system (0.67 [95% CI, 0.62–0.72] vs. 0.63 [95% CI, 0.58–0.78], *P* = 0.631).

### Performance of the nomogram in predicting the cancer death risk of patients

**Fig. 3:**
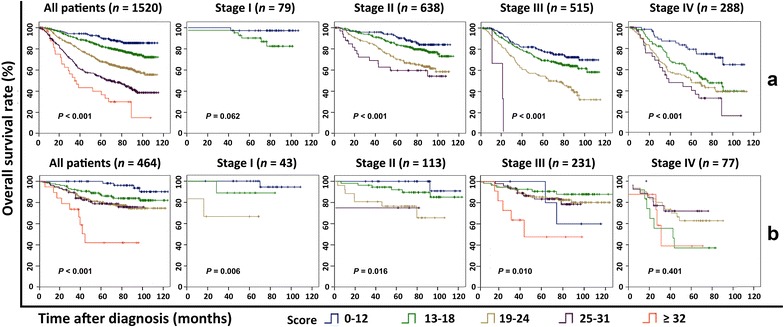
Kaplan–Meier OS curves of non-metastatic NPC patients in each stage stratified by the scores of risk (0–12, 13–18, 19–24, 25–31, and ≥32) in the internal validation cohort (**a**) and IMRT cohort (**b**) (pooled log-rank test)

We scored the cancer death risk of every patient and classified the patients into five subgroups with distinct prognoses. The 5 year OS rates of the five risk subgroups with risk scores of 0–12, 13–18, 19–24, 25–31, and ≥32 were 90, 82, 68, 54, and 40%, respectively. When patients in the primary cohort were grouped according to their respective TNM stages (I, II, III, and IV), the stratification by risk scores resulted in significant differences in Kaplan–Meier OS curves for patients in each stage group, except for patients in stage I (Fig. 3a). As for the IMRT cohort, this stratification also resulted in significant differences in OS, except for patients in stage IV (Fig. 3b).

## Discussion

In the present study, we evaluated the prognostic values of several known predictors for survival of NPC patients. The association between the clinicopathologic factors and prognosis of NPC patients has been well established in previous studies [14–21] and was then confirmed in the present study. Notably, the serum level of LDH ranked third in contributing to the prognosis of NPC patients, which followed the N and T stages. This finding called attention to the importance of the baseline LDH, which might indicates the tumor activity and tendency for metastasis in NPC patients, especially in combination with ALP level [22]. Based on these results, we established a nomogram that combined 12 significant clinical factors to visualize the prediction of the prognosis of NPC patients.

With respect to the validation methods, both internal validation and external validation are appropriate. However, internal validation, such as cross-validation and bootstrap resampling, has a theoretical probability of overinterpretation [9]. Therefore, external validation is more appropriate for examining the model applicability, e.g., using a data set from other institutions or a validation set by the data-splitting method from the same institution, especially when the sample size is large [7, 13]. In this study, we calibrated the nomogram with a validation set and an IMRT cohort from the same institution that were independent from the training set. First, the high level of agreement between the expected and actual observed OS in the splitting internal validation set demonstrated the accuracy of the nomogram. In addition, discrimination was revealed by the significantly higher c-index (an index similar to the area under the receiver-operating characteristic curves in the diagnostic test) of the nomogram compared with the TNM staging system. The distinct risk stratification of patients within the same stage illustrated the additional prognostic values of incorporating other factors.

As it conforms more to the tumor shape and minimizes the toxicity to surrounding normal tissues, IMRT has been increasingly applied and has even replaced conventional radiotherapy (CRT) in recent years [23]. Considering that patients in the training set on which the nomogram was built predominantly received CRT, we retrieved a pure cohort that was treated with IMRT. An underestimation of OS was observed for the nomogram. Additionally, the predictive accuracy (c-index) of the nomogram decreased slightly in the IMRT cohort, which was in contrast to that of the TNM staging system. This might be a result of overfit bias, which is inevitable when developing a nomogram. In addition, the patient selection periods for the primary and IMRT datasets did not completely overlap. To the best of our knowledge, the patients who underwent CRT and IMRT primarily differed in their adverse events and short-term outcomes, but they did not necessarily differ in the OS [24]. Moreover, the included variables have no mechanical association with the radiotherapy modality. Therefore, we cautiously concluded that this nomogram could project to the IMRT population according to the acceptable predictive power and accuracy.

Several previous models established for NPC also showed improved prognosis prediction of the TNM staging system by adding some functional factors [25–27]. However, the practical use of these models was restricted because they only provided stratification of risks at a population level without offering an association between the individual patient and his/her corresponding OS. In contrast, the nomogram we developed in this study could serve as both a scoring system and a visualized predicting tool, which could help physicians rapidly match a patient with his/her expected OS through a simple calculation in clinical practice. In addition, this nomogram could assist in the clinical study design, balancing the prognostic background between different arms, especially for non-randomized data. This function shares a similar rationale with propensity score-matched analysis [28].

This nomogram developed for NPC patients assists clinicians in many aspects. Still, there are some limitations. First, the retrospective nature of the database might result in bias and calls for prospective validation of the model. Second, we have not yet validated the model using an external dataset from other institutes, although we are seeking further collaborations. Third, although the EBV-DNA copy number might be a strong prognostic factor, we failed to incorporate it into the nomogram because EBV-DNA was not routinely tested in our center until 2007 [29].

In conclusion, we developed and validated a novel nomogram for non-metastatic NPC patients. This nomogram provides a more accurate and precise prediction for the OS compared with the TNM staging system. This nomogram could help clinicians with decision-making and study design. In addition, this nomogram could be used to evaluate the prognosis of the IMRT population.
